# Outcomes Related to Percutaneous Nephrostomies (PCN) in Malignancy-Associated Ureteric Obstruction: A Systematic Review of the Literature

**DOI:** 10.3390/jcm10112354

**Published:** 2021-05-27

**Authors:** Francesca J. New, Sally J. Deverill, Bhaskar K. Somani

**Affiliations:** 1 Department of Urology, University Hospital Southampton, Southampton SO16 6YD, UK; frankiejnew@gmail.com; 2 Department of Urology, Queen Alexandra Hospital, Portsmouth PO6 3LY, UK; s.deverill@doctors.org.uk

**Keywords:** prostate cancer, nephrostomy, quality of life, survival, decision making

## Abstract

Background: Malignant ureteric obstruction occurs in a variety of cancers and has been typically associated with a poor prognosis. Percutaneous nephrostomy (PCN) can potentially help increase patient longevity by establishing urinary drainage and treating renal failure. Our aim was to look at the outcomes of PCN in patients with advanced cancer and the impact on the patients’ lifespan and quality of life. Materials and Methods: A literature review was carried out for articles from 2000 to 2020 on PCN in patients with advanced malignancies, using MEDLINE, EMBASE, Scopus, CINAHL, Cochrane Library, clinicaltrials.gov, and Google Scholar. All English-language articles reporting on a minimum of 20 patients who underwent PCN for malignancy-associated ureteric obstruction were included. Results: A total of 21 articles (1674 patients) met the inclusion criteria with a mean of 60.2 years (range: 21–102 years). PCN was performed for ureteric obstruction secondary to urological malignancies (*n* = −633, 37.8%), gynaecological malignancies (*n* = 437, 26.1%), colorectal and GI malignancies (*n* = 216, 12.9%), and other specified malignancies (*n* = 205, 12.2%). The reported mean survival times varied from 2 to 8.5 months post PCN insertion, with an average survival time of 5.6 months, which depended on the cancer type, stage, and previous treatment. Conclusions: Patients with advanced malignancies who need PCN tend to have a survival rate under 12 months and spend a large proportion of this time in the hospital. Although the advent of newer chemotherapy and immunotherapy options has changed the landscape of managing advanced cancer, decisions on nephrostomy must be balanced with their survival and quality of life, which must be discussed with the patient.

## 1. Introduction

Malignancy-associated ureteric obstruction occurs in a variety of pelvic cancers, often as a late manifestation, which can be secondary to locally advanced disease or nodal metastases. Treatment consists of various options ranging from ureteric stent insertion (retrograde or antegrade), to percutaneous nephrostomy (PCN), to other forms of urinary diversion. While these procedures can help to improve renal function, they also risk complications and can have a profound effect on the quality of life (QoL). Stenting can consign the patient to stent symptoms (which may include frequency, urgency, pain, haematuria, and dysuria), and regular stent changes (typically every 6–12 months) under a general anaesthetic but is generally believed to be better for QoL than long-term PCN, although give the underlying disease this might be challenging [1].

Unfortunately, in the context of locally advanced pelvic cancers, there are often scenarios whereby a patient will start with a retrograde ureteric stent (RUS), but subsequently, as this fails, it necessitates PCN insertion. In the event that a RUS change or drainage fails, the decision to proceed with PCN often marks disease progression. Without treatment of malignant ureteric obstruction, the patient will deteriorate over time with symptoms of uraemia, fluid overload, electrolyte disturbances, flank pain, urinary infections, reduction in alertness, renal failure, and subsequent death [2]. Patients with advanced malignancies, who present with acute renal failure (ARF) due to malignant ureteric obstruction, are often poor surgical and/or anaesthetic candidates, and therefore PCN, which can be done under local anaesthesia (LA), is often preferred. Similarly, it is not always possible to insert primary retrograde stents in the context of locally advanced pelvic malignancies [3,4,5].

Percutaneous nephrostomy has a high rate of technical success; however, periprocedural complications can occur. These may include sepsis, bleeding or vascular injury, perirenal haematoma, and injury to surrounding structures such as colon, liver, and lung [3]. Furthermore, PCN can block, dislodge, develop line or component fracture, become infected, or colonised with bacteria, and patients can develop skin reactions, cellulitis, or abscesses [3]. Such complications can result in multiple readmissions to hospital, often needing a change in PCN, which can also significantly impact their QoL [1]. Emergency readmissions also happen if the PCN falls out completely, needing a new nephrostomy placement as a matter of urgency [6]. Patients with advanced cancers who develop infections secondary to nephrostomy are at a high risk of deterioration, especially if they are receiving immunosuppression such as chemotherapy or immunotherapy.

Most studies looking at malignancy-associated ureteric obstruction cover an extremely heterogenous population, with multiple different aetiologies and presentations. Treating malignant ureteric obstruction is an ever-changing landscape, and as newer cancer treatments become available, this continues to evolve. We aimed to review the quality of evidence available to date in this group of patients, establishing outcomes of PCN in malignancy-associated ureteric obstruction, assessing the risk of complications, life expectancy, QoL and potential indicators of favourable versus poorer outcomes.

## 2. Materials and Methods

### 2.1. Study Population

Population: Adults with malignancy-associated ureteric obstruction.

Intervention: Percutaneous nephrostomy.

Comparator: Not applicable for this study.

Outcome: Life expectancy, QoL, and outcomes related to PCN.

### 2.2. Inclusion Criteria

Studies reporting on patients with advanced malignancies with ureteric obstruction.

English-language studies reporting on a minimum of 20 patients.

### 2.3. Exclusion Criteria

PCN insertion for benign disease.

Studies that included primary ureteric stenting as the only treatment option.

Case reports, laboratory studies, or review articles.

### 2.4. Search Strategy and Study Selection

The systematic review was performed as per the Cochrane guidelines and the Preferred Reporting Items for Systematic Reviews and Meta-analyses (PRISMA) checklist [7]. The database searched were MEDLINE, EMBASE, Scopus, CINAHL, Cochrane Library, clinicaltrials.gov and Google Scholar from January 2000 to December 2020. The search terms included ‘Nephrostomy’, ‘percutaneous nephrostomy’, ‘PCN’, ‘urinary drainage’, ‘stent’, ‘ureteric stent’, ‘prostate’, ‘ovarian’, ‘cervical’, ‘bowel’, ‘malignancies, malignancy or cancer’, and ‘pelvic, gynaecological, colorectal, urological’. Boolean operators (AND, OR) were used with the above search terms to refine the search. Two reviewers (S.D. and F.N.) independently identified all the studies that matched the inclusion criteria and any discrepancies were resolved by consensus with the senior author (BKS).

### 2.5. Data Extraction and Analysis

The primary outcome measures were complications after PCN, time spent in the hospital after PCN, and survival times after their first PCN. Secondary outcomes were QoL after PCN and differences in outcomes based on the cancer sub-type. Information was collected on the year of publication, type of malignancy, patient demographics, and outcomes of PCN. Data were collected using Microsoft Excel 2019 (version 19.0). A narrative review was done due to heterogeneity of the studies and data available.

## 3. Results

### 3.1. Literature Search and Included Studies

**Figure 1:**
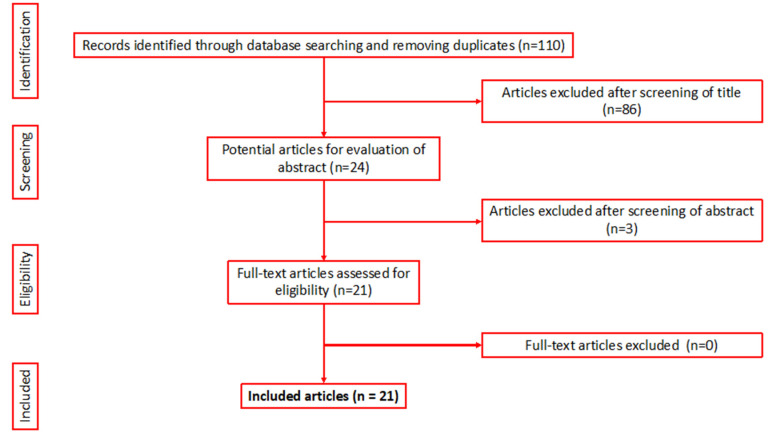
PRISMA flowchart of the included articles.

After an initial search of 110 articles, 21 studies (1674 patients) met the inclusion criteria for the final review (Figure 1) [3,6,8,9,10,11,12,13,14,15,16,17,18,19,20,21,22,23,24,25,26]. A full breakdown of the patient demographics can be seen in Table 1.

### 3.2. Patient Characteristics

There were 1674 patients with a mean age of 60.2 years (range: 21–102 years), although two studies did not state the mean or median age [6,7], and two studies stated the median age [8,9]. The majority of studies were retrospective in nature (*n* = 17), with one prospective study [8] and four where the type of study was not specified (Table 1) [10,11,12,13].

PCN was performed for ureteric obstruction secondary to urological malignancies (*n* = 633, 37.8%), gynaecological malignancies (*n* = 437, 26.1%), colorectal and gastro-intestinal (GI) malignancies (*n* = 216, 12.9%), and other specified malignancies (*n* = 205, 12.2%) (Table 1) [13]. Fourteen studies documented the length of survival post nephrostomy insertion for the different cancer subtypes [3,8,9,11,12,15,16,17,18,19,20,21,22,23].

### 3.3. Primary Outcomes

#### 3.3.1. Survival Times after PCN

**Figure 2:**
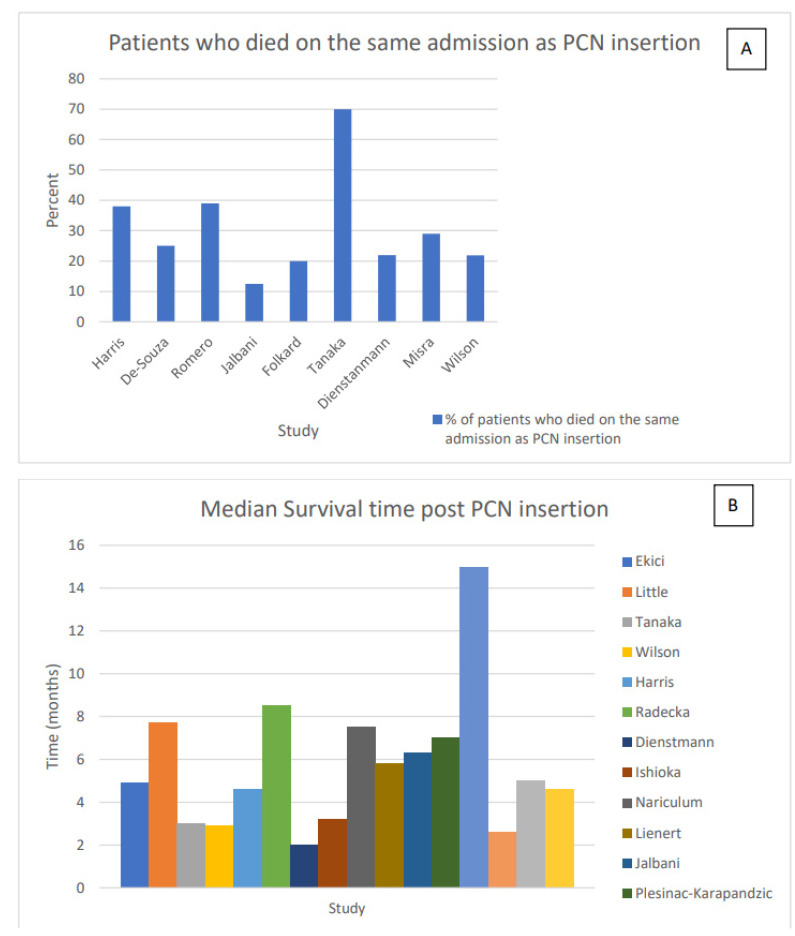
(**A**): Patients who died on the same admission as nephrostomy (PCN) insertion (**B**): Median survival post PCN insertion.

The reported mean survival time varied from 2.6 to 8.5 months post initial PCN insertion, with an average survival time of 5.9 months (Figure 2, Table 1). Five studies documented median survival time as 5.2 months (range: 2–7 months) [8,9,10,11,22], and three did not document the survival time post PCN insertion [6,13,24].

Romeo et al. [18] documented the survival times post PCN insertion with 40% dead at 6 months and a further 24.4% at 1 year, while Aravantious documented that 67% of the patients were dead within 6 months of a PCN insertion [21] (Table 1). A prostate cancer study by Nariculam and colleagues in 2009 found that the overall mean time to death post PCN was 7.5 months, but if patients developed ureteric obstruction while already on hormones, the mean survival decreased to 4.5 months. In the context of newly diagnosed and hormone-naïve patients, the survival increased to a mean of 16 months (range: 1–38 months) [23]. Similarly, Harris et al. found that survival was longer for the hormone-naïve group (226.5 days) when compared to 100.2 days in the castrate-resistant prostate cancer group [20].

In the context of bladder cancer, Ekici et al. looked at 23 patients with malignant ureteric obstruction due to bladder cancer, including patients with new diagnosis of locally advanced disease, disease recurrence post cystectomy, and those with metastatic disease. There was a mean survival of 4.9 months (range: 1–14 months). Eighteen (78%) died of disease progression or irreversible renal failure after malignant ureteric obstruction during the study period [17].

Romero et al. found that prognosis was worse in patients over 52 years old and in patients with bladder cancer or hormone refractory prostate cancer, rather than cervical cancer, but patient numbers were small (*n* = 43), so this may not be generalisable [18]. Misra et al. reported a median survival post PCN insertion as only 78 days (range: 4–1137 days) and also described that the subset of bladder cancer patients seemed to do more poorly [12]. In contradiction to these findings, Jalbani described an improved median survival in urogenital malignancies (bladder and prostate) of 350 days (range: 150–700 days) when compared to non-urogenital malignancies, except lymphoma (gynaecological, colorectal, breast, and gallbladder cancers) where the median survival was only 25 days (range: 7–80 days) [8].

Folkard et al. found that the average survival time post PCN was 139 days, and there was no significant difference between the cancer subgroups in terms of survival time post nephrostomy. They also showed that a greater improvement in renal function did not improve the survival time. A large proportion of their patients (65.7%) did not undergo further oncological treatment post PCN as they became too frail for it [25].

#### 3.3.2. Prognostic Indicators

Alawneh et al. found that the factors associated with a shorter survival time were type of malignancy, bilateral hydronephrosis, serum albumin <3.5 mg/dL, presence of metastases, ascites, or pleural effusion. Survival was better if patients had only one risk factor, with median survival 17.6 months vs. 1.7 months if four risk factors were present. The overall 12-month survival in their paper was 33.7% [9]. Ishioka [15] found that the factors associated with a poorer prognosis included colorectal cancer, three or more events related to metastatic disease, degree of hydronephrosis, and serum albumin <3 g/dL.

Lienert et al.’s [10] prognostic indicators were consistent with previously discussed studies; a serum albumin <3 mg/dL and three or more events related to dissemination of cancer were factors significantly associated with shorter mean survival. Moreover, a sodium <135 mEq/L was found to be a significant prognostic factor. In this study, degree of hydronephrosis was not found to be a significant prognostic factor.

Nariculum et al. [23] showed that the mean survival for newly diagnosed patients (hormone-naïve) was 16 months (range: 1–38 months), compared to patients who developed ureteric obstruction while on hormones, where the mean survival was only 4.5 months (range: 10 days to 17 months). This was also shown by Harris et al., who showed that hormone-naïve patients survived longer at 226.5 days, compared to 114.3 days in hormone-responsive groups and 100.2 days in the hormone-resistant group. Another prognostic factor was the failure of renal function to improve despite nephrostomies, and if the post-procedure urea and creatinine went below 15 mmol/L and below 250 μmol/L, respectively, then the mean survival time was 192.4 days, but if the renal function did not improve, then the mean survival was only 30.7 days [20].

Romero et al. showed that the poor prognostic factors in their study were age above 52 years and patients with bladder and hormone refractory prostate cancer [18]. Misra also showed that patients with bladder cancer had a worse prognosis [12]. In contrast, Radecka et al. [3] and Jalbani et al. [8] showed an improved survival in patients with bladder cancer. De Souza et al. demonstrated that the finding of hypotension unrelated to septic symptoms was a risk factor for progression to death [24].

#### 3.3.3. Complications of PCN

Nineteen studies commented on the complication rates (Table 2). The overall complication rate ranged from 7% to 87%. The majority of the complications were minor, including urinary tract infection, haematuria, skin infection, malposition/dislodgement of PCN tubing and self-limiting fever. There was, however, a reasonably high rate of kinking, dislodgement, or loss of nephrostomy requiring reinsertion. There were some major complications described, including two patients who required a nephrectomy due to severe infection and peri-renal abscesses [9].

McDevitt et al. specifically looked at the number of routine vs. emergency PCN changes. Out of 87 PCN exchanges or reinsertions, only 33% were routine and 67% were for emergency reasons such as infection, obstruction, displacement, or mechanical complications [6].

Insertion of the initial PCN has good rates of technical success. Aravantinos et al. reported a 2.5% failure rate, with no serious complications, a minor temperature rise of 55%, and a transfusion rate of 2.9%; however, they commented on pre-existing anaemia, and therefore this may not be related to the PCN insertion itself. They also reported that a small proportion of patients (4.4%) needed staged a second nephrostomy tube due to persistent uraemia despite a unilateral nephrostomy tube [21].

#### 3.3.4. Bilateral vs. Unilateral PCN

One point of interest was whether in order to improve QoL in patients with bilateral hydronephrosis secondary to malignant ureteric obstruction, a unilateral nephrostomy was sufficient. Thirteen studies commented on whether they inserted unilateral or bilateral nephrostomies. In prostate cancer, one study reported that the mean survival for unilateral nephrostomy patients was better (157.6 days) than for those who required bilateral nephrostomies, whether they were placed simultaneously or staged [20]. This could be due to the fact that they also demonstrated that a worse prognosis is linked with bilateral hydronephrosis. In one study of mixed malignancies, 92% of the patients had bilateral hydronephrosis and their aim was to trial unilateral PCN. Only 4.4% patients required a second-stage nephrostomy due to persistent uraemia despite having a unilateral nephrostomy [21].

#### 3.3.5. Quality of Life after PCN

There are no validated questionnaires specifically looking at QoL with nephrostomies in cancer patients [27]. A wide range of methods for determining quality of life with a nephrostomy were used throughout the studies. Aravantinos et al. [21] used the QoL questionnaire EORTC-QLC-C30 [28] and found that QoL improved at 1 month, and of the different cancer subgroups, it was better in the prostate cancer subgroup. Wilson et al. used the criteria of Grabstald and McPhee to define ‘useful quality of life’ and found 17/32 (53.1%) did not fulfil such criteria, and the subgroup of bladder cancer patients had poorer outcomes [19]. Misra used the Watkinson criterion (if the patient was able to leave hospital for 6 weeks or more), finding that 64% would have satisfied this criterion [12]. In the studies that measured QoL, only around half of the patients achieved an adequate QoL post PCN insertion.

#### 3.3.6. In-Hospital Stay after PCN

The time spent in hospital following PCN insertion was highly variable and poorly reported (Table 1). Romero found that the percentage of lifetime left that was spent in hospital was 17.7%, and 57.7% of those discharged from hospital had to be readmitted (either due to disease progression or complications from PCN) [18]. Wilson reported a mean hospital stay of 29 days from PCN insertion to death or end of study period, and each patient was readmitted an average of 1.6 times until death [19]. Misra reported a median hospital stay post PCN of 23 days (range: 3–89), with 29% of a patient’s end of life spent in hospital [12]. Folkard had a mean hospital stay of 14 days post PCN; however, 39% of the patients were readmitted, and 20% spent their remaining life in hospital [25].

Many patients with advanced malignancies die in hospital despite PCN insertion, and nine studies reported the percentage of patients who died on the same hospital admission as their PCN was placed [8,12,16,18,19,20,22,24,25]. The mean percentage of patients who died on the same hospital admission as their PCN insertion was 30.8% and ranged from 12.5% to 70% (Figure 2).

## 4. Discussion

### 4.1. Findings of Our Study

The mean survival time varied from 2.6 to 8.5 months post initial PCN insertion across the studies, with an average survival time of 5.9 months (Figure 2, Table 1). The majority of studies agreed that hormone-naïve prostate cancer had a longer survival time post PCN insertion, whereas bladder cancer, cervical cancer, and hormone refractory prostate cancer all had shortened life expectancies. Poor prognostic indicators throughout the studies were patients who had already undergone cancer treatment, presence of multiple metastasis, type of cancer, degree of hydronephrosis, and a low serum albumin concentration. The number of days spent in hospital post PCN insertion were high (Table 1) and a third of the patients (range: 12.5–70%) died on the same admission while they were admitted to hospital (Figure 2).

### 4.2. Patient Counselling

The ethics of palliative urinary decompression have been debated, and many factors must be taken into account, such as the type and stage of malignancy, the ability for further palliative treatment, patient’s quality and quantity of life along with their preference. Malignant ureteric obstruction from pelvic malignancies often presents a significant treatment dilemma for urologists. While PCN insertion is relatively safe, patients with advanced malignancies tend to have a higher risk of PCN-related complications (Table 2) and spend a large proportion of their time in hospitals. PCNs should only be pursued after thoughtful counselling regarding further treatment options and likely disease prognosis.

### 4.3. Quality of Life

There are no validated questionnaires specifically looking at QoL with nephrostomies in cancer patients [27]. A wide range of methods for determining QoL with a nephrostomy were used throughout the studies, ranging from whether the patient ever left hospital at all, to whether they left hospital for 6 weeks or more (Watkinson criteria [29]), to scoring them on four criteria; of little or no pain, full mental capacity, few complications related to PCN insertion, and the ability to return home (Grabstald and McPhee criteria [19]), to using EORTC-QLC-C30 questionnaires [28]. It is difficult to ascertain whether QoL is worse after PCN insertion due to the procedure, or the progression of the cancer; hence a standardised questionnaire would be useful in ascertaining this and could aid patients in making the decision on whether or not to proceed with a nephrostomy [27].

### 4.4. Costs of Replacement of PCN

McDevitt et al. looked at patients who had nephrostomies placed for malignant ureteric obstruction, and the causes of PCN exchanges during the follow-up period. There were 87 exchanges performed, and of those, 29/87 (33.3%) were routine elective changes, but 58/87 (66.7%) were unplanned and due to complications, such as infection (21/87, 33%), obstruction (23/87, 26%) or mechanical complications (14/87, 16%). The cost of emergency exchange vs. routine exchange was modelled to be higher, and they therefore hypothesised that decreasing the length of time to routine exchange from 90 days to 60 days would decrease the amount of readmissions for emergency exchange or replacement, which would decrease the overall cost [6].

### 4.5. Conversion of PCN to Ureteric Stents

In some cases, where PCN has been inserted primarily, it may be possible to convert it to an indwelling ureteric stent, usually via antegrade stenting. Wilson and colleagues reported that in 34.4% of cases, they were able to have PCN converted to an indwelling stent [19], and Misra et al. reported that 56% of all PCNs were subsequently antegradely stented and rendered nephrostomy free [12]. Folkard reported that 65% of PCNs were converted to stents.

### 4.6. Limitations

Almost all of the studies were retrospective, and with historic data, which made it difficult to apply them to today’s cancer patients with recent advances in cancer treatment. These studies cover a heterogeneous population with some having a variety of different primary cancers, while others focus on a single cancer type, which makes interpretation difficult. As novel immunotherapy and chemotherapy options emerge, the ability to predict prognosis is more guarded, and newer information is needed to aid decision making. There were no data from situations where patients presented with hydronephrosis and the decision was not to perform PCN, and how their QoL and length of life compared to those with PCN.

Since the studies reported included a wide time interval (from 2003 to 2020), it should be appropriate to take into account that some malignancies have improved treatment options with potential benefits to prognosis and quality of life. For example, in colorectal cancer, starting from 2004 several drugs have been introduced (cetuximab, bevacizumab, and panitumumab) with advantage on cancer-specific survival. Similar improvements have been reported in prostate cancer from 2011 with new hormone-based therapies (abiraterone and enzalutamide) in metastatic castration-resistant patients, and from 2015 in metastatic hormone-sensitive patients. This treatment may also affect the quality of life and the number of days spent in hospital. Moreover, in selected cases, the option of a new treatment line can justify the insertion of ureteric stent or nephrostomy.

The retrospective nature of the included papers with different inclusion criteria makes it liable to selection bias and hence difficult to draw meaningful comparisons. Given that almost a third of the patients died on the same hospital admission as their PCN insertion suggests that a high number of reported PCNs were performed for palliative reasons. The decision on nephrostomy would have to be individualised for a given patient and must take into account their medical condition and underlying disease status.

### 4.7. Areas of Future Research

Prognosis of patients with malignant ureteric obstruction is mostly dependent on further treatment strategies. In recent years, there has been a big leap in oncological therapies, many of which are reliant on good renal function. In many situations now, where there is malignant ureteric obstruction, a patient may still have further options for palliative chemotherapy, immunotherapy or novel hormone therapies. However, if there are no options in reserve, the prognosis is poor with or without nephrostomies, and end-of-life care should be discussed with the patient and relatives, rather than proceeding with invasive interventions that have no impact on disease progression. Complications and death due to locally invasive cancer should be weighed against complications and death due to uraemia.

## 5. Conclusions

There is little doubt about the benefits of percutaneous nephrostomy for patients with a new diagnosis of disease, allowing improvement of renal function to allow staging investigations. However, in patients in the end stages of their cancer, PCN insertion should only be placed after thoughtful counselling regarding further treatment options available and disease prognosis, given that with advanced malignancies, many patients have a short life expectancy, spending most of their time in the hospital with a poor quality of life.

## Figures and Tables

**Table 1:** Demographics, survival, cancer sub-type and time spent in the hospital.

Author/Year Published	Review Period	Mean Age (Range), Years	Total Number of Patients	Time Spent in Hospital after PCN	Survival Time after Insertion of PCN (Mean)	Malignancy Type	Number of Patients (Subgroup)	Breakdown of Survival per Cancer Type Post PCN Insertion (Months)
Ekici et al. [17], 2001	1987–2000	55 (25–76)	23	ND	4.9 months	Bladder	23	4.9
Little et al. [26], 2003	ND	69 (50–87)	31	46% of remaining life in hospital	7.7 months	Bladder	16	ND
						Prostate	8	
						Colorectal	4	
						Gynaecological	3	
Tanaka et al. [16], 2004	1991–2003	69.2	33	70% remained in hospital post PCN	3 months	Urological	8	3.0
						Gynaecological	8	3.0
						Colorectal	8	5.5
						Upper GI	8	1.5
						Lung	1	2.5
Romero et al. [18], 2005	2000–2004	52	43	17% of their remaining life (58% readmission rate)	40% survival at 6 months, 24.2% survival at 1 year	Urological	15	40% survival at 6 months/10% at 1 year
						Gynaecological	28	44.6% survival at 6 months/38.45% at 1 year
Wilson et al. [19], 2005	1998–2001	68.1 (42–84)	32	29 days (81% readmission rate)	2.9 months	Urological	17	2.4
						Gynaecological	7	6.9
						Colorectal	7	4.3
						Breast	1	27.1
Harris et al. [20], 2006	2001–2004	75.9 (65–89)	26	51 days (Mean)	4.6 months	Prostate	26	2.9 months
Carrafiello et al. [13], 2006	2003–2006	65.7 (32–102)	201	ND	ND	ND	201	ND
Radecka et al. [3], 2006	1998–2002	73.1 (51–97)	151	ND	8.5 months	Prostate	55	6.9
						Bladder	43	17.6
						Gynaecological	11	31.2
						Colorectal	16	4.3
						other	26	10.1
Aravantinos et al. [21]. 2007	1996–2003	63 (40–86)	270	ND	67% of patients died in first 6 months post PCN	Bladder	54	8–270 days
						Prostate	54	22–723 days
						Gynae	54	7–269 days
						Colorectal	54	9–272 days
						other	54	8–280 days
Dienstmann et al. [22], 2008	2002–2006	44 (26–67)	50	22% of patients remained in hospital post PCN	2 months (median)	Cervical	50	2 months
Ishioka et al. [15], 2008	1995–2007	57 (31–85)	140	ND	3.2 months	Urological	13	ND
						Gynaecological	36	
						Colorectal	34	
						other	57	
Nariculam et al. [23], 2009	1998–2006	71 (51–85)	25	ND	7.5 months	Prostate	25	7.5 months
Lienert et al. [10], 2009	2005–2007	71 (36–91) median	49	ND	5.8 months (median)	Urological	33	ND
						Gynaecological	5	
						Colorectal	6	
						other	5	
Jalbani et al. [8], 2010	2004–2006	ND, (range 21–70)	40	ND	6.3 months (median)	Urological	15	14.3
						Gynaecological	17	11.3
						Colorectal	3	1.2
						other	5	ND
Plesinac-Karapandzic et al. [11], 2010	1996–2006	51 (28–85) median	117	ND	7 months (median)	Gynaecological	117	7 months
Malik et al. [14], 2010	2001–2009	68.67 (53–85)	28	ND	15 months	Prostate cancer	28	15 months
Misra et al. [12], 2013	2008	75.1 (54–87)	22	29% of life in hospital (100% readmission rate)	2.6 months	Urological	18	33% survival at 6 months
						Gynaecological	2	100% survival at 6 months
						Colorectal	2	0% survival at 6 months
Alawneh et al. [9], 2016	2009–2013	Not reported	211	ND	5 months (median)	Urological	122	5.5
						GI	61	5.2
						Other	28	3.6
De Souza et al. [24], 2016	2010–2012	48.2	45	ND	ND	Cervical cancer	45	ND
McDevitt et al. [6], 2017	2011–2013	48 (21–79)	57	ND	ND	Cervical	26	ND
						Colorectal	6	
						Prostate	6	
						Bladder	4	
						Lymphoma	3	
						Ovarian	3	
						other	9	
Folkard et al. [25], 2020	2015–2018	68.8 (30–93)	105	Median post procedure 14 days (1–104 days)	4.6 months	Bladder	32	ND
						Prostate	18	
						Colorectal	16	
						Gynaecological	25	
						Other	8	

**Table 2:** Complications of percutaneous nephrostomy (PCN) insertion.

Author	Type of Complication and %	Overall Complications
Ekici et al. [17]	Occlusion/dislodgement/malposition 30%	30%
Little et al. [26]	Occlusion/dislodgement/malposition 13%	13%
Tanaka et al. [16]	Infection/sepsis 54%	54%
Romero et al. [18]	Nephrectomy 5%	42%
Wilson et al. [19]	Occlusion/dislodgement/malposition 46.2%	46.2%
Carrafiello et al. [13]	Occlusion/dislodgement/malposition 17.3% Haematuria 1%	18.3%
Radecka et al. [3]	Occlusion/dislodgement/malposition 7%	7%
Aravantinos et al. [21]	Infection/sepsis 55% Transfusion 2.9%	47.9%
Dienstmann et al. [22]	Infection/sepsis 32% Occlusion/dislodgement/malposition 18% Death 4% Pain 2% Haematuria 2%	58%
Ishioka et al. [15]	Infection/sepsis 13% Occlusion/dislodgement/malposition 19% Haematuria 8%	40%
Nariculam et al. [23]	Infection/sepsis 4% Occlusion/dislodgement/malposition 12% Haematuria 8%	24%
Lienert et al. [10]	Infection/sepsis 22.4% Occlusion/dislodgement/malposition 63% Haematuria 2%	87%
Jalbani et al. [8]	Infection/sepsis 7.5% Occlusion/dislodgement/malposition 37.5% Haematuria 5%	50%
Plesinac-Karapandzic et al. [11]	Infection/sepsis 39.2% Occlusion/dislodgement/malposition 37.6%	76.8%
Malik et al. [14]	-	4–25%
Misra et al. [12]	-	27%
De Souza et al. [24]	Infection/sepsis 42% Occlusion/dislodgement/malposition 15.5% Perirenal haematoma <5%	62.5%
McDevitt et al. [6]	Infection/sepsis 24% Occlusion/dislodgement/malposition 42.5%	66.5%
Folkard et al. [25]	-	39%

## References

[1] Bigum L.H., Spielmann M.E., Juhl G., Rasmussen A. (2014). A qualitative study exploring male cancer patients’ experiences with percutaneous nephrostomy. Scand. J. Urol..

[2] Kouba E., Wallen E.M., Pruthi R.J. (2008). Management of ureteral obstruction due to advanced malignancy: Optimising therapeutic and palliative outcomes. J. Urol..

[3] Radecka E., Magnusson A. (2004). Complications associated with percutaneous nephrostomies. A retrospective study. Acta Radiol..

[4] Wah T.M., Weston M.J., Irving H.C. (2004). Percutaneous nephrostomy insertion: Outcome data from a prospective multi-operator sudy at a UK training centre. Clin. Radiol..

[5] Patel U., Hussain F.F. (2004). Percutaneous Nephrostomy of Nondilated Renal Collecting Systems with Fluoroscopic Guidance: Technique and Results. Radiology.

[6] McDevitt J.L., Acosta-Torres S., Zhang N., Hu T., Odu A., Wang J., Xi Y., Lamus D., Miller D.S., Pillai A.K. (2017). Long-Term Percutaneous Nephrostomy Management of Malignant Urinary Obstruction: Estimation of Optimal Exchange Frequency and Estimation of the Financial Impact of Patient Compliance. J. Vasc. Interv. Radiol..

[7] Liberati A., Altman D.G., Tetzlaff J., Mulrow C., Gøtzsche P.C., Ioannidis J.P.A., Clarke M., Devereaux P.J., Kleijnen J., Moher D. (2009). The PRISMA statement for reporting systematic reviews and meta-analyses of studies that evaluate healthcare interventions: Explanation and elaboration. BMJ.

[8] Jalbani M.H., Deenari R.A., Dholia K.R., Oad A.K., Arbani I.A. (2010). Role of percutaneous nephrostomy (PCN) in malignant ureteral obstruction. J. Pak. Med. Assoc..

[9] Alawneh A., Tuqan W., Innabi A., Al-Nimer Y., Azzouqah O., Rimawi D., Taqash A., Elkhatib M., Klepstad P. (2016). Clinical Factors Associated With a Short Survival Time After Percutaneous Nephrostomy for Ureteric Obstruction in Cancer Patients: An Updated Model. J. Pain Symptom Manag..

[10] Lienert A., Ing A., Mark S. (2009). Prognostic factors in malignant ureteric obstruction. BJU Int..

[11] Plesinac-Karapandzic V., Masulovic D., Markovic B., Djuric-Stefanovic A., Plesinac S., Vucicevic D., Milovanovic Z., Milosevic Z. (2010). Percutaneous nephrostomy in the management of advanced and terminal-stage gynecologic malignancies: Outcome and complications. Eur. J. Gynaecol. Oncol..

[12] Misra S., Coker C., Richenberg J. (2013). Percutaneous nephrostomy for ureteric obstruction due to advanced pelvic malignancy: Have we got the balance right yet?. Int. Urol. Nephrol..

[13] Carrafiello G., Laganà D., Mangini M., Lumia D., Recaldini C., Bacuzzi A., Marconi A., Mira A., Cuffari S., Fugazzola C. (2006). Complications of percutaneous nephrostomy in the treatment of malignant ureteral obstructions: Single–centre review. La Radiol. Med..

[14] Malik M.A., Mahmood T., Khan J.H., Hanif A., Bajwa I.A. (2010). Experience of percutaneous nephrostomy (PCN) in advanced ca prostate. PJMHS.

[15] Ishioka J., Kzgeyama Y., Inoue M., Higashi Y., Kihara K. (2008). Prognostic Model for predicting survival after palliative urinary diversion for utereral obstruction: Analysis of 140 cases. J. Urol..

[16] Tanaka T., Yanase M., Takatsuka K. (2004). Clinical course in patients with percutaneous nephrostomy for hydronephrosis associated with advanced cancer. Hinyokika Kiyo. Acta Urol. Jpn..

[17] Ekici S., Şahin A., Özen H. (2001). Percutaneous Nephrostomy in the Management of Malignant Ureteral Obstruction Secondary to Bladder Cancer. J. Endourol..

[18] Romero F.R., Broglio M., Pires S.R., Roca R.F., Guibu I.A., Perez M.D. (2005). Indications for percutaneous nephrostomy in patients with obstructive uropathy due to malignant Urogenital neoplasias. Int. Braz. J. Urol..

[19] Wilson J.R., Urwin G.H., Stower M.J. (2005). The role of percutaneous nephrostomy in malignant ureteric obstruction. Ann. R. Coll. Surg. Engl..

[20] Harris M.R.E., Speakman M.J. (2006). Nephrostomies in obstructive uropathy; how should hormone resistant prostate cancer patients be managed and can we predict who will benefit?. Prostate Cancer Prostatic Dis..

[21] Aravantinos E., Anagnostou T., Karatzas A.D., Papakonstantinou W., Samarinas M., Melekos M.D. (2007). Percutaneous nephrostomy in patients with tumors of advanced stage: Treatment dilemmas and impact on clinical course and Qaulity of life. J. Endourol..

[22] Dienstmann R., Pinto C.D.S., Pereira M.T., Small I.Á., Gil Ferreira C. (2008). Palliative Percutaneous Nephrostomy in Recurrent Cervical Cancer: A Retrospective Analysis of 50 Consecutive Cases. J. Pain Symptom Manag..

[23] Nariculam J., Murphy D.G., Jenner C., Sellars N., Gwyther S., Gordon S.G., Swinn M.J. (2009). Nephrostomy insertion for patients with bilateral ureteric obstruction caused by prostate cancer. Br. J. Radiol..

[24] De Souza A.C.P., Souza A.N., Kirsztajn R., Kirsztajn G.M. (2016). Cervical cancer: Renal Complications and survival after percutaneous nephrostomy. Rev. Assoc. Med. Bras..

[25] Folkard S.S., Banerjee S., Menzies-Wilson R., Reason J., Psallidas E., Clissold E., Al-Mushatat A., Chaudhri S., Green J.S.A. (2020). Percutaneous nephrostomy in obstructing pelvic malignancy does not facilitate further oncological treatment. Int. Urol. Nephrol..

[26] Little B., Ho K.J., Gawley S., Young M. (2003). Use of nephrostomy tubes in ureteric obstruction from incurable malignancy. Int. J. Clin. Pract..

[27] New F., Deverill S., Somani B.K. (2018). Role of percutaneous nephrostomy in end of life prostate cancer patients: A systematic review of the literature. Cent. Eur. J. Urol..

[28] Aaronson N.K., Ahmedzai S., Bergman B., Bullinger M., Cull A., Duez N.J., Filiberti A., Flechtner H., Fleishman S.B., De Haes J.C. (1993). The European Organization for Research and Treatment of Cancer QLQ-C30: A Quality-of-Life Instrument for Use in International Clinical Trials in Oncology. J. Natl. Cancer Inst..

[29] Watkinson A., A’Hern R., Jones A., King D., Moskovic E. (1993). The role of percutaneous nephrostomy in malignant urinary tract obstruction. Clin. Radiol..

